# Impact of cervical osteoarthritis on quality of life after free flap reconstruction in head and neck cancer

**DOI:** 10.3389/fonc.2025.1630458

**Published:** 2025-09-10

**Authors:** Yili Li, Wei Du, Xu Zhang, Junhui Yuan, Yibao Sun, Zhe Shao, Shuang Chen, Yaojun Dai, Xiaoguang Zhou, Yong Yang, Wei Mei

**Affiliations:** ^1^ Department of Spinal Surgery, Zhengzhou Orthopedic Hospital, Henan, Zhengzhou, China; ^2^ Department of Head Neck and Thyroid, The Affiliated Cancer Hospital of Zhengzhou University and Henan Cancer Hospital, Zhengzhou, China; ^3^ Department of Radiology, The Affiliated Cancer Hospital of Zhengzhou University and Henan Cancer Hospital, Zhengzhou, China

**Keywords:** head and neck squamous cell carcinoma, cervical osteoarthritis, quality of life, free flap, propensity score matching

## Abstract

**Background:**

Head and neck squamous cell carcinoma (HNSCC) often requires free flap reconstruction, which involves extensive cervical manipulation. Concurrently, cervical osteoarthritis is increasingly prevalent due to modern lifestyle factors. This study investigates the impact of cervical osteoarthritis on quality of life (QoL) in HNSCC patients undergoing free flap reconstruction.

**Methods:**

A retrospective analysis of HNSCC patients who underwent free flap reconstruction was conducted. Patients were stratified by cervical osteoarthritis status and assessed using the Neck Disability Index (NDI), EORTC QLQ-C30, and QLQ-HN35 questionnaires at preoperative, postoperative, and follow-up intervals (3, 6, and 12 months post-adjuvant therapy). Propensity score matching (1:1) adjusted for confounding factors.

**Results:**

A total of 60 patients were analyzed. At baseline, cervical osteoarthritis was associated with lifting dysfunction (p=0.004) and nausea/vomiting (p<0.05). Postoperatively, transient impairments in driving, sleeping, and swallowing were noted in the osteoarthritis group, particularly within 3 months (p<0.05). Persistent fatigue was reported, but emotional function scores were superior in this group (p<0.05). No significant differences in QoL were observed based on osteoarthritis subtype. Response rates declined over time, primarily due to cancer recurrence.

**Conclusions:**

Cervical osteoarthritis correlates with transient postoperative functional impairments and persistent fatigue but does not adversely affect long-term QoL. Emotional resilience in these patients suggests adaptive coping mechanisms. These findings underscore the need for targeted preoperative counseling and rehabilitation to address short-term challenges while leveraging patients’ psychosocial strengths. This study provides critical insights for optimizing patient-centered care in HNSCC reconstruction.

## Introduction

Head and neck squamous cell carcinoma (HNSCC) ranks as the sixth most common malignancy among solid tumors, with over half of patients presenting with lymph node metastasis at initial diagnosis (1). Standard treatment typically involves primary tumor resection and neck dissection (2). Given the critical functional roles of the head and neck region—including appearance, swallowing, mastication, and speech—free flap reconstruction is frequently employed to optimize functional recovery. However, this approach necessitates extensive cervical manipulation. Additionally, adjuvant radiotherapy (RT) or chemoradiation (CRT), while essential for improving oncologic outcomes, often adversely impacts quality of life (QoL) (3).

The rising prevalence of sedentary lifestyles and prolonged poor posture, exacerbated by widespread use of electronic devices, has contributed to an increasing incidence of cervical osteoarthritis (4). This trend is also observed in HNSCC patients, whose therapeutic interventions—such as surgical reconstruction and adjuvant therapies—may accelerate the progression of preexisting cervical degenerative disease, potentially leading to cervical myelopathy or other severe neurologic complications. To date, this clinical concern has been scarcely documented, with only a single case report describing a 63-year-old man without prior cervical spondylosis who developed quadriplegia on postoperative day 4 following free fibula osteocutaneous flap reconstruction for gingivo-buccal SCC. MRI confirmed severe cervical myelopathy, necessitating decompressive laminectomy; despite rehabilitation, his functional recovery remained limited (5).

Given this unmet clinical need, we conducted a prospective study to evaluate the impact of cervical osteoarthritis on QoL in HNSCC patients undergoing free flap reconstruction.

## Patients and methods

### Ethical approval

This study was approved by Henan Cancer Hospital Institutional Research Committee, and written informed consent for medical research was obtained from all patients before starting the treatment. All methods were performed in accordance with the relevant guidelines and regulations.

### Study design

To fulfill our objectives, a retrospective analysis of prospectively collected data was undertaken. A cohort of 335 patients who underwent free flap reconstruction for primary HNSCC at a tertiary hospital from January 2020 to December 2024 were enrolled. This observational study included all participants, except for 10 who were excluded due to flap necrosis and 5 due to prior neck trauma. The exclusion of these cases may introduce selection bias, as flap necrosis is often associated with compromised wound healing or vascular insufficiency, which could independently affect functional recovery and QoL outcomes. Similarly, patients with prior cervical trauma may have preexisting structural or neurological deficits that confound the assessment of osteoarthritis-related disability. However, these exclusions were necessary to isolate the specific impact of cervical osteoarthritis on postoperative QoL without the compounding effects of surgical complications or unrelated cervical pathology. The remaining patients were requested to complete the Neck Disability Index (NDI), the EORTC QLQ-C30, and QLQ-H&N35 questionnaires at various intervals: preoperatively, prior to adjuvant therapy, and at 3 months, 6 months, and one year post-adjuvant therapy. Data pertaining to demographics, treatment modalities, and follow-up were meticulously gathered.

### Variable definition

Data on cervical osteoarthritis were comprehensively collected during the initial consultation. The condition was diagnosed through a combination of clinical assessment and radiological evaluation. Clinically, patients were evaluated for symptoms such as neck pain, radiculopathy, myelopathy, or autonomic dysfunction. Radiologically, cervical spine imaging (X-ray, MRI, or CT) was performed to confirm degenerative changes, including disc space narrowing, osteophyte formation, facet joint arthritis, or spinal cord compression. Based on these findings, cervical osteoarthritis was categorized into subtypes: cervical spondylosis without neurological involvement (CSNI), cervical radiculopathy (CR), cervical myelopathy (CM), vertebrobasilar insufficiency (VI), sympathetic cervical spondylosis (SCS), and mixed type (MT) (6). Body Mass Index (BMI) was deemed normal within the range of 18.5 to 24.9 (7). Cancer staging was determined according to the 8th edition of the AJCC system. The head and neck regions were delineated as oral cavity/oropharynx (OC/OP) and larynx/hypopharynx (LA/HP). Neck dissections were classified into functional neck dissection (FND) and radical neck dissection (RND), with the latter involving the resection of the sternocleidomastoid muscle, internal jugular vein, and accessory nerve.

### Questionnaire

The NDI comprises 10 items, each scored from 0 (no disability) to 5 (unable to perform the activity), yielding a maximum total score of 50. The assessed domains include pain intensity, personal care, lifting, reading, headaches, concentration, work, driving, sleeping, and recreation. Total scores of 0-4, 5-14, 15-24, 25-34, and ≥35 correspond to none/minimal, mild, moderate, severe, and complete disability, respectively (8).

The QLQ-C30 is a comprehensive 30-item questionnaire evaluating general cancer-related symptoms and functional status. The items are grouped into functional scales (higher scores indicate better functioning) such as physical, role, emotional, cognitive, and social functioning, and symptom scales (higher scores denote worse symptoms) including fatigue, nausea/vomiting, pain, dyspnea, insomnia, appetite loss, constipation/diarrhea, along with a global health scale. Raw scores are linearly transformed to a 0–100 scale (9).

The QLQ-HN35 questionnaire, consisting of 35 items, assesses HNSCC-specific issues. It includes symptom scales (higher scores indicate worse symptoms) such as pain, swallowing problems, senses, speech problems, social eating, social contact, sexuality, and single-item symptoms (10).

### Treatment

All patients underwent curative primary site excision and neck dissection followed by immediate microvascular free flap reconstruction performed by a dedicated head and neck surgical team. In patients with cervical osteoarthritis, specific intraoperative neck positioning protocols were implemented to minimize cervical spine stress. These included use of neutral head positioning with supportive padding, and avoidance of hyperextension or excessive rotation during intubation and surgery. Patients with severe cervical osteoarthritis underwent preoperative evaluation by an orthopedic or neurosurgical specialist. Recommendations from these consultations guided perioperative management. For OC/OP cancers, levels I-III/IV were dissected for cN0 necks and I-IV/V for cN+ necks. For LA/HP cancers, levels II-IV were dissected for cN0 necks and II-IV/V for cN+ necks.

All patients underwent adjuvant radiotherapy (RT) using image-guided intensity-modulated radiation therapy (IG-IMRT) with standardized protocols. The prescribed doses were: 60-66 Gy in 30-33 fractions (2 Gy/fraction, 5 fractions weekly) to the high-risk volume (tumor bed and involved nodal regions), and 54-56 Gy to elective nodal regions (levels II-IV for oropharyngeal cancers, levels II-V for hypopharyngeal cancers). Patients with extranodal extension or positive margins received simultaneous integrated boost to 66 Gy in 30 fractions with weekly cisplatin (40 mg/m²). Rigorous organ-at-risk constraints were implemented, including spinal cord Dmax < 45 Gy, cervical spine mean dose < 35 Gy (with particular attention to C5-C7 segments), and avoidance of circumferential cord irradiation. Treatment planning incorporated deformable image registration to account for postoperative anatomical changes, and daily cone-beam CT verification ensured positional accuracy. These parameters followed NCCN guidelines while being adapted for patients with preexisting cervical osteoarthritis, including additional immobilization precautions to minimize neck extension during treatment (11). Patients were followed up every 3 months for the first two years, every 6 months for the subsequent three years, and annually from the fifth year onwards.

### Statistical analysis

Clinicopathologic variables were compared between patients with and without cervical osteoarthritis using the Chi-square test. Propensity score matching was conducted based on significant factors at a 1:1 ratio. The primary outcome variable was Quality of Life (QoL). The impact of cervical osteoarthritis on QoL was assessed via the Mann-Whitney U test. All statistical analyses were executed using the R 3.4.3 software package, with statistical significance set at a p-value of less than 0.05.

## Results

### Baseline data

A total of 320 patients were enrolled, with a mean age of 50 ± 10 years, comprising 210 males and 110 females. Among them, 129 were identified as smokers and 104 as drinkers. Comorbid conditions, including diabetes, adrenal cortical insufficiency, and others, were present in 121 patients. A normal BMI was observed in 135 individuals. Cancer staging revealed 216 patients at stages II/III and 104 at stage IV. FND and RND were performed in 227 and 93 patients, respectively, with the majority undergoing unilateral neck dissection. Additionally, 82 patients underwent fibula flap reconstruction. All patients received adjuvant radiotherapy, with 96 also undergoing chemotherapy.

The mean operative time was 8.2 ± 1.5 hours (range 6-11 hours), including an average ischemia time of 72 ± 12 minutes for flap transfer. Prophylactic tracheostomy was performed in 87.5% of cases (280/320 patients). Nutritional support included mandatory nasogastric tube placement intraoperatively was performed in all patients. Flap selection was tailored to minimize cervical mobility restriction, utilizing fibula flaps for bony defects and soft tissue flaps for mucosal coverage. Postoperative care included 72-hour cervical collar immobilization for osteoarthritis patients and early shoulder range-of-motion exercises initiated on postoperative day 2 to prevent adhesive capsulitis (**Table 1**).

**Table 1:** Baseline data of the patients with or without cervical osteoarthritis (CO) before propensity score matching.

Variable	Total (n=320)	CO (n=30)	No CO (n=290)	P^
Age				
<50	164	18	146	
≥50	156	12	144	0.314
Sex				
Male	210	15	195	
Female	110	15	95	0.058
Smoker				
No	191	24	167	
Yes	129	6	123	0.017
Drinker				
No	216	26	190	
Yes	104	4	100	0.019
Basic disease				
No	199	22	177	
Yes	121	8	113	0.186
BMI				
<18.5	97	8	89	
18.5-24.9	135	14	121	
≥25	88	8	80	0.856
Primary site^!^				
OC/OP	227	22	205	
LA/HP	93	8	85	0.761
Cancer stage				
II/III	216	19	197	
IV	104	11	93	0.609
Neck dissection type*				
FND	227	20	207	
RND	93	10	83	0.588
Neck dissection side				
Unilateral	216	17	199	
Bilateral	104	13	91	0.183
Flap type^&^				
RFF/ALT	238	26	212	
Fibula	82	4	78	0.126
Adjuvant therapy^%^				
T	224	24	200	
CRT	96	6	90	0.209
Operation time (h)	8.2 ± 1.5	8.5 ± 1.6	8.1 ± 1.5	0.142
Flap ischemia time (min)	72 ± 12	75 ± 14	71 ± 11	0.108
Prophylactic tracheostomy				
No	40	5	35	
Yes	280	25	255	0.782

^comparison between the CO and no CO groups using the Chi-square test.

!OC/OP, oral cavity/oropharynx; LA/HP, larynx/hypopharynx.

*FND, functional neck dissection; RND, radical neck dissection.

&RFF, radial forearm flap; ALT, anterolateral thigh flap.

%RT, radiotherapy; CRT, chemoradiation.

Thirty patients were diagnosed with cervical osteoarthritis, predominantly characterized as non-smokers (p=0.017) or non-drinkers (p=0.019). These factors were incorporated into a propensity score matching with a 1:1 ratio, resulting in a final cohort of 60 patients (30 in each group) for QoL analysis. No significant differences in clinicopathologic factors were observed between the two groups (all p>0.05, **Table 2**).

**Table 2:** Baseline data of the patients with or without cervical osteoarthritis (CO) after propensity score matching.

Variable	Total (n=60)	CO (n=30)	No CO (n=30)	P^
Age				
<50	35	18	17	
≥50	25	12	13	0.793
Sex				
Male	30	15	15	
Female	30	15	15	1.000
Smoker				
No	48	24	24	
Yes	12	6	6	1.000
Drinker				
No	52	26	26	
Yes	8	4	4	1.000
Basic disease				
No	43	22	21	
Yes	17	8	9	0.774
BMI				
<18.5	15	8	7	
18.5-24.9	30	14	16	
≥25	15	8	7	0.816
Primary site^!^				
OC/OP	44	22	22	
LA/HP	16	8	8	1.000
Cancer stage				
II/III	39	19	20	
IV	21	11	10	0.787
Neck dissection type*				
FND	38	20	18	
RND	22	10	12	0.592
Neck dissection side				
Unilateral	35	17	18	
Bilateral	25	13	12	0.793
Flap type^&^				
RFF/ALT	51	26	25	
Fibula	9	4	5	1.000
Adjuvant therapy^%^				
RT	46	24	22	
CRT	14	6	8	0.542
Operation time (h)	8.4 ± 1.6	8.5 ± 1.6	8.2 ± 1.6	0.487
Flap ischemia time (min)	74 ± 13	75 ± 14	73 ± 12	0.562
Prophylactic tracheostomy				
No	9	5	4	
Yes	51	25	26	1.000

^comparison between the CO and no CO groups using the Chi-square test.

!OC/OP, oral cavity/oropharynx; LA/HP, larynx/hypopharynx.

*FND, functional neck dissection; RND, radical neck dissection.

&RFF, radial forearm flap; ALT, anterolateral thigh flap.

%RT, radiotherapy; CRT, chemoradiation.

### Response rate

The response rate was 100% preoperatively and prior to adjuvant therapy. However, it declined to 90% at 3 months, 83.3% at 6 months, and 73.3% at 12 months post-adjuvant therapy in the cervical osteoarthritis group. In the non-cervical osteoarthritis cohort, the corresponding rates were 92.4%, 86.2%, and 81.7%. The decrement in response rates was attributed to cancer recurrence.

### NDI questionnaire

**Figure 1:**
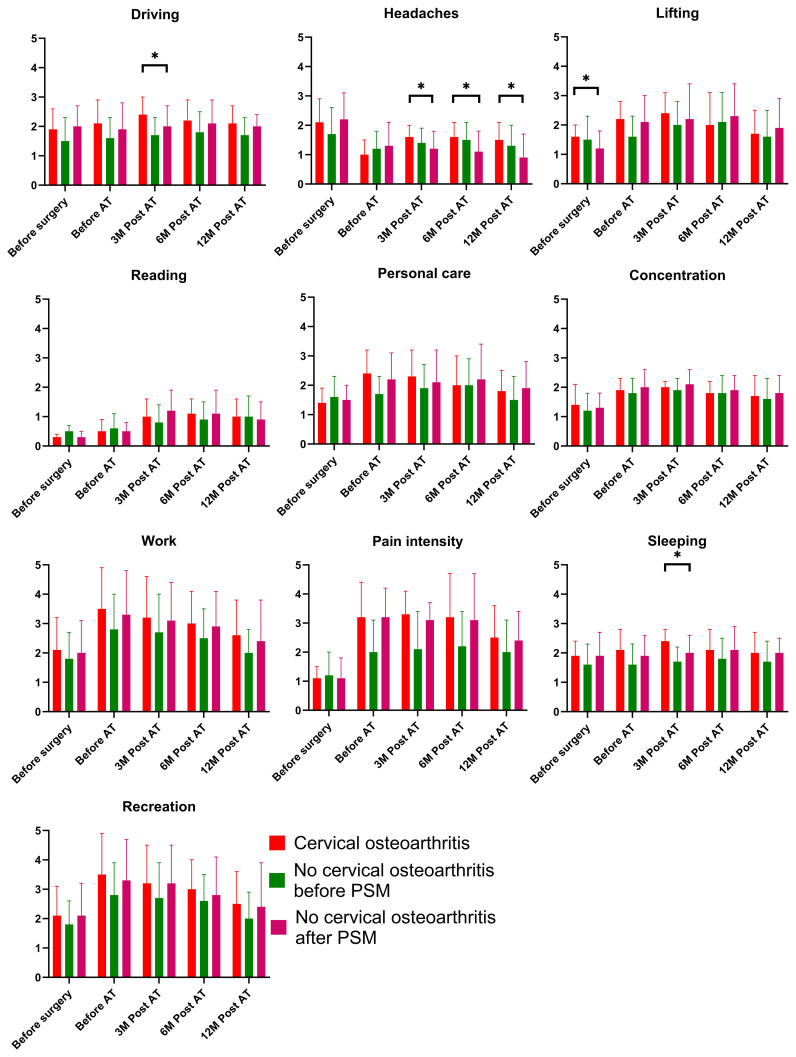
Comparison of the Neck Disability Index results in patients with and without cervical osteoarthritis. * is indicator of "p<0.05".

At baseline, all neck functioning domains, except for lifting (p=0.004), exhibited comparable results between the cervical osteoarthritis and non-cervical osteoarthritis groups after PSM (all p > 0.05). At subsequent time points, five significant differences were identified. Patients with cervical osteoarthritis demonstrated notably higher scores for headaches at 3, 6, and 12 months post-adjuvant therapy, driving at 3 months post-adjuvant therapy, and sleeping at 3 months post-adjuvant therapy (**Figure 1**). No other significant disparities were observed between the two groups.

### QLQ-C30 questionnaire

**Figure 2:**
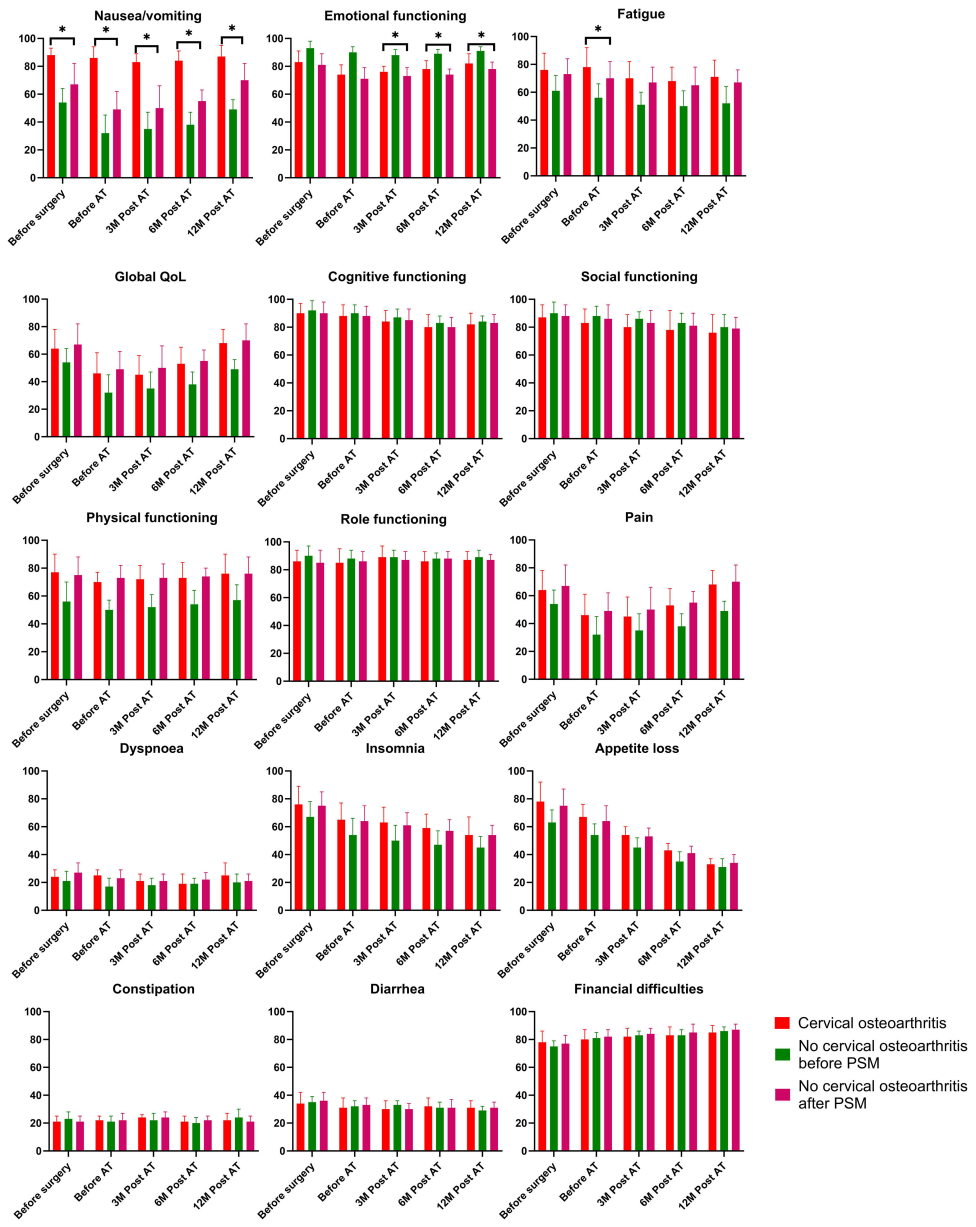
Comparison of the EORTC QLQ-C30 results in patients with and without cervical osteoarthritis. * is indicator of "p<0.05".

At baseline, all items, except for nausea/vomiting, yielded similar scores between the cervical osteoarthritis and non-cervical osteoarthritis groups after PSM. Regarding the functioning scale, patients with cervical osteoarthritis exhibited superior emotional function scores at 3, 6, and 12 months post-adjuvant therapy compared to those without cervical osteoarthritis (**Figure 2**). In terms of symptom items, the cervical osteoarthritis group reported greater fatigue before adjuvant therapy, and higher nausea/vomiting scores at all time points. No other significant differences were noted between the two groups.

### QLQ-HN35 questionnaire

**Figure 3:**
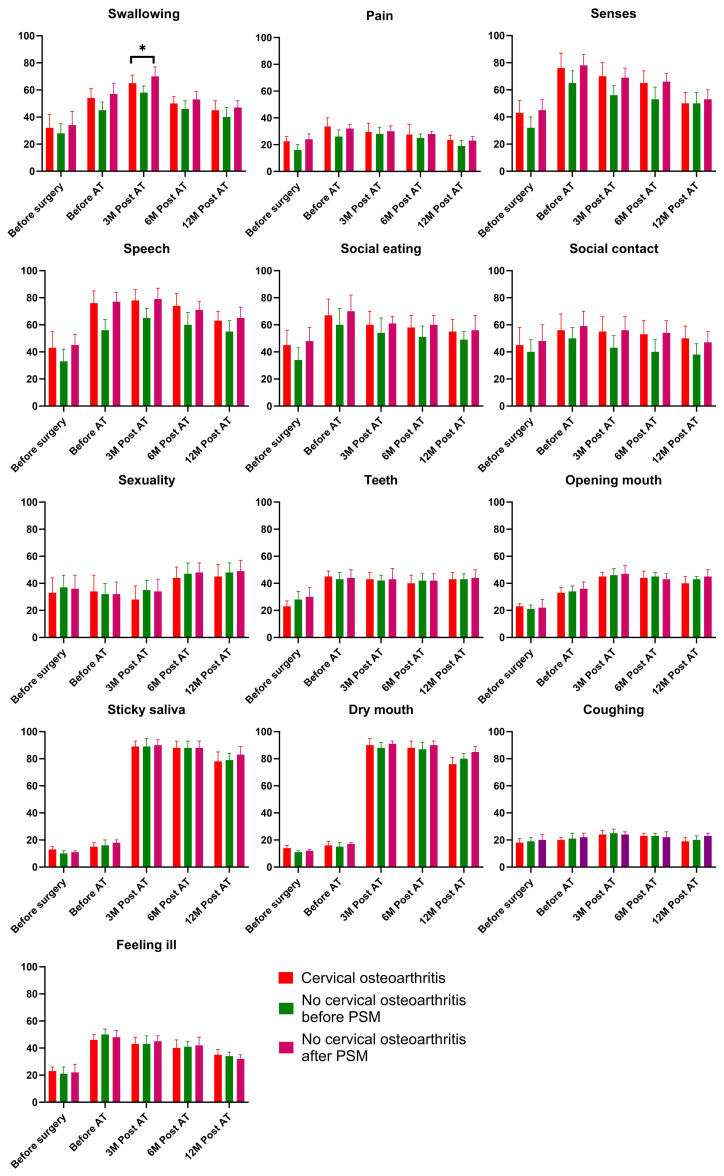
Comparison of the QLQ-H&N35 results in patients with and without cervical osteoarthritis. * is indicator of "p<0.05".

At baseline, all items scored similarly between the cervical osteoarthritis and non-cervical osteoarthritis groups after PSM. However, patients with cervical osteoarthritis demonstrated higher swallowing scores at 3 months post-adjuvant therapy compared to those without cervical osteoarthritis. No other significant differences were observed between the two groups (**Figure 3**).

### Subgroup analysis

The classification of cervical osteoarthritis revealed 17 patients diagnosed with CSNI and 13 cases identified as CR. Notably, the two groups exhibited comparable scores across all items in the three administered questionnaires (**Supplementary Figures 1**–**3**).

## Discussion

Our study revealed three key findings: (1) cervical osteoarthritis was associated with baseline lifting dysfunction and nausea/vomiting that resolved postoperatively; (2) post-reconstruction, these patients experienced transient impairments in driving, sleeping, and swallowing along with persistent fatigue, yet maintained superior emotional function; and (3) osteoarthritis subtype had no significant impact on quality of life. As the first investigation of cervical osteoarthritis’s effects on QoL following HNSCC reconstruction, these findings provide valuable insights for clinical counseling and patient expectations.

The head and neck region’s functional complexity means HNSCC treatments often impair critical functions, necessitating evidence-based rehabilitation. Prior studies highlight these challenges: one involving 61 survivors linked longer surgeries and hospital stays to increased depression and speech difficulties (12), while another of 58 patients with RFF flap reconstruction reported preserved QoL (>70% on functioning scales) but persistent fatigue, sexual dysfunction, and oral complications (13). However, these studies were limited by their focus on survivors alone, omitting preoperative comparisons and preexisting neck dysfunction—gaps our study addresses.

Cervical osteoarthritis, a degenerative disorder of ligaments and facet joints, primarily affects the C5-C6 and C6-C7 levels, though high cervical involvement also occurs. Typical symptoms include neck pain [prevalence: 0.4-86.8% (14), affecting ~349 million globally (15)], radicular numbness, nausea/vomiting, and vertigo, with severity varying by lesion location. In HNSCC patients, free flap reconstruction combined with adjuvant radiotherapy accelerates cervical osteoarthritis progression through radiation-induced soft tissue fibrosis (16) and chemotherapy-enhanced oxidative stress (17). This explains both the transient 3-month functional declines (coinciding with peak RT toxicity) and persistent fatigue in our cervical osteoarthritis cohort, mirroring findings in non-cancer cervical osteoarthritis patients receiving neck irradiation. Our study uniquely demonstrates that cervical osteoarthritis correlates with lifting dysfunction and nausea/vomiting - findings consistent with known cervical degeneration effects (18). Notably, we observed no significant pain score elevation in osteoarthritis patients, likely because tumor-related pain (common in T3/4 HNSCC) overshadowed degenerative pain.

In the subsequent post-surgery period, at least three interesting points were noted. Firstly, a history of cervical osteoarthritis correlated with transient impairments in driving, sleeping, and swallowing after free flap reconstruction, and these dysfunction tended to occur before adjuvant therapy or within 3 months after adjuvant therapy. On one hand, these short-term impairments may reflect the combined impact of surgical disruption and preexisting cervical degeneration. For instance, reduced neck mobility from cervical osteoarthritis could compromise swallowing mechanics post-flap reconstruction, while pain-related sleep disturbances might persist until healing stabilizes. On the other hand, the 3-month window was not long enough for either natural recovery or treatment-related adaptation, suggesting synergistic effects of radiation/chemotoxicity and cervical osteoarthritis-related biomechanical constraints, neural rehabilitation or tissue remodeling always need six months (19). Secondly, baseline cervical osteoarthritis symptoms resolved, but new dysfunctions emerged post-surgery. Potential explanation might be attributed by surgical trauma unmasking latent cervical osteoarthritis vulnerability or compensatory mechanisms failing under new physical demands (20). Thirdly, the observed dichotomy—where cervical osteoarthritis patients reported persistent fatigue but superior emotional function—may reflect distinct adaptive pathways. Chronic fatigue could stem from prolonged cervical spine dysfunction, sleep disturbances, or the cumulative burden of pain and adjuvant therapy, all of which are known sequelae of cervical osteoarthritis. Conversely, their sustained emotional resilience might be attributed to psychological adaptation mechanisms, such as ‘response shift’, wherein patients recalibrate their QoL expectations after enduring chronic cervical degeneration prior to cancer diagnosis. Alternatively, cervical osteoarthritis patients may engage more proactively with psychosocial support during treatment, having already navigated long-term disability. This finding aligns with prior studies in chronic pain populations, where emotional well-being often decouples from physical symptoms due to coping strategies or reframing of illness perceptions. Clinically, this underscores the need to address fatigue (e.g., via graded exercise or sleep hygiene interventions) while leveraging these patients’ emotional strengths to enhance recovery.

This study highlights several actionable steps to optimize care for HNSCC patients with cervical osteoarthritis undergoing free flap reconstruction. First, preoperative counseling should explicitly address the likelihood of transient post-operative impairments in driving, sleeping, and swallowing—particularly during the first 3 months—while reassuring patients that these symptoms typically resolve. Second, early rehabilitation programs should be tailored to this population, incorporating cervical mobility exercises, swallow therapy, and sleep hygiene education to mitigate anticipated dysfunction. Third, clinicians should consider routine screening for fatigue in cervical osteoarthritis patients during long-term follow-up, as this may persist even after physical recovery. Paradoxically, the observed emotional resilience in cervical osteoarthritis patients suggests an opportunity to leverage their adaptive coping strategies through peer support programs or psychological interventions. Finally, multidisciplinary teams should prioritize clear communication about recovery timelines to align patient expectations with the predicted trajectory—initial physical challenges followed by gradual improvement. These measures could enhance functional outcomes while capitalizing on this population’s unique psychosocial strengths.

While propensity score matching helped adjust for confounding variables between groups, our study has several limitations. First, the lack of a true control group without cervical osteoarthritis prevents direct comparison to the general HNSCC population undergoing free flap reconstruction. Although we compared patients with and without cervical osteoarthritis, a non-osteoarthritis control group matched for all surgical and adjuvant therapy parameters would have strengthened the causal interpretation of our findings. This limitation reflects the pragmatic challenges of studying this specific comorbidity in oncologic surgery populations. Future prospective studies could address this by including matched controls from multicenter cohorts.

In summary, while cervical osteoarthritis was associated with transient postoperative impairments in driving, sleeping, and swallowing, these functional limitations typically resolved within the early recovery period. Persistent fatigue alongside sustained emotional resilience highlight the complex interplay between physical and psychosocial adaptation in this patient population. Importantly, our results demonstrate that cervical osteoarthritis subtype has minimal impact on long-term quality of life, suggesting that preoperative counseling should focus more on functional expectations than diagnostic classification. These findings underscore the importance of targeted rehabilitation strategies and patient-centered communication to optimize recovery in cervical osteoarthritis patients undergoing complex head and neck reconstruction.

## Data Availability

The original contributions presented in the study are included in the article/**Supplementary Material**. Further inquiries can be directed to the corresponding authors.

## Supplementary material

Supplementary Figure 1 Comparison of the Neck Disability Index results in patients with different types of cervical osteoarthritis.

Supplementary Figure 2 Comparison of the EORTC QLQ-C30 results in patients with different types of cervical osteoarthritis.

Supplementary Figure 3 Comparison of the QLQ-H&N35 results in patients with different types of cervical osteoarthritis.
